# The Effect of a Name-Based Mask Rationing Plan in Taiwan on Public Anxiety Regarding a Mask Shortage During the COVID-19 Pandemic: Observational Study

**DOI:** 10.2196/21409

**Published:** 2021-01-22

**Authors:** Yu-Lin Tai, Hsin Chi, Nan-Chang Chiu, Cheng-Yin Tseng, Ya-Ning Huang, Chien-Yu Lin

**Affiliations:** 1 Hsinchu MacKay Memorial Hospital Department of Pediatrics and Infectious Disease Hsinchu Taiwan; 2 MacKay Children's Hospital Department of Pediatrics and Infectious Disease Taipei Taiwan; 3 MacKay Medical College Department of Medicine New Taipei Taiwan; 4 China Medical University Graduate Institute of Chinese Medicine, School of Chinese Medicine Taichung Taiwan

**Keywords:** coronavirus, COVID-19, novel coronavirus, SARS-CoV-2, mask, rationing, Taiwan, anxiety, mental health, observational, crisis, plan

## Abstract

**Background:**

The COVID-19 pandemic is a severe global health crisis. Wearing a mask is a straightforward action that can be taken, but shortage of stock and equity of allocation were important issues in Taiwan. Furthermore, increased anxiety leading to the stockpiling of masks has been common during the pandemic.

**Objective:**

We aim to summarize the name-based mask rationing plan implemented in Taiwan and explore the public’s perceived anxiety about mask shortages.

**Methods:**

The government of Taiwan took action to control the supply and allocation of face masks. We summarize the timeline and important components of the mask rationing plan. A survey that aimed to investigate the overall response to the mask rationing plan was answered by 44 participants.

**Results:**

The mask rationing plan was implemented in late January 2020. Daily production capacity was increased from 2 million masks to 16 million masks in April 2020. People could buy 9 masks in 14 days by verification via their National Health Insurance card. Digital face mask availability maps were created. Moreover, the mask plan safeguarded the purchase of masks and resulted in decreased anxiety about a mask shortage (4.05 [SD 1.15] points; 72.7% [n=32] of participants answered “agree” or “strongly agree”). The majority of people felt that the mask plan was satisfactory (4.2 [SD 0.92] points; 79.5% [n=35] of participants answered “agree” or “strongly agree”).

**Conclusions:**

We found that the unique name-based mask rationing plan allowed for control of the production and supply of masks, and contributed to the appropriate allocation of masks. The mask rationing plan not only provided the public with physical protection, but also resulted in reduced anxiety about mask shortages during the pandemic.

## Introduction

The COVID-19 pandemic has become a severe global crisis and there were more than 20 million cases as of late August 2020 [1]. This disease is highly contagious with protean clinical manifestations, making it difficult to prevent disease spread [2-4]. The Taiwanese government implemented several strategies early on and Taiwan had a relatively controllable situation [1,5]. As of late August, Taiwan had a total of 496 cases (approximately 20 cases per million residents), with 7 mortalities [6]. Taiwan’s success in combating COVID-19 captured our attention and mask use in the public was believed to play a crucial role in the battle against COVID-19 [7-9].

Wearing masks to protect against viral transmission is straightforward but attitudes toward mask use varied across countries [10]. The recommendations regarding mask wearing varied across time and as the severity of the pandemic changed [6,10]. Some people felt discomfort due to having something covering their faces and were afraid of asphyxia; as a result, some people were not willing to wear a mask. Furthermore, the protective effects of wearing masks were doubted in some areas. The protective effectiveness of wearing a mask during a mass gathering was investigated and a relative risk of 0.89 was found [11]. Wearing a mask was found to result in a large reduction of infection risk (adjusted odds ratio 0.15) in a recent systematic review [7]. Although controversies related to wearing masks existed, wearing a mask was believed to be protective during the pandemic [6].

Wearing a mask is common in Asian countries; prior to the COVID-19 pandemic, people wore masks in public places to prevent infection. Due to the sudden, unexpected, and overwhelming pandemic, people panic-bought masks in Taiwan. Increased anxiety about mask shortages led to people stockpiling masks. Thus, the shortage of mask storage, soaring prices, and equity of allocation were important issues in the early phase of the COVID-19 pandemic. The government of Taiwan took action to control the supply and allocation of face masks; this unique mask plan was believed to have greatly contributed to the success of the battle against COVID-19 in Taiwan [5,9,12]. Furthermore, mask-buying surged since the pandemic and anxiety about inadequate mask supply was also noted. In addition to medical illness, mental health issues might arise in response to the COVID-19 pandemic [13-16]. Reallocation and a guarantee of available masks may reduce panic buying during the pandemic. We aim to summarize the unique name-based mask rationing plan in Taiwan and study whether the mask rationing system might have contributed to a reduction in public anxiety about mask shortages during the COVID-19 pandemic.

## Methods

Our study was approved by the ethical committee of MacKay Memorial Hospital, Taipei, Taiwan (registration number 20MMHIS140e). We summarize here the strategies undertaken by the government of Taiwan, as well as details of the name-based mask rationing plan, taken from the website of the Centers for Disease Control, Taiwan (CDC) [5]. As the face mask production rate increased, the rationing plan evolved over time.

To investigate the potentially psychogenic impacts of the mask plan, we also conducted a simple survey with a 5-point scale questionnaire that aimed to investigate the overall response to the mask rationing plan. Taiwanese residents aged >18 years were freely recruited at the entrance of our hospital. They were able to read and write Mandarin. The questionnaire was anonymous and not related to medical services. There were 10 simple questions using plain language and it took approximately 1-2 minutes to finish the questionnaire (Table 1). Question 1 explored the respondent’s attitude toward mask use. Questions 3, 4, and 8 investigated the number of masks required by the respondent. Questions 5 and 9 were regarding the prices of masks. Questions 2 and 6 surveyed the respondent’s perceived anxiety about a potential mask shortage. Finally, questions 7 and 10 investigated the waiting time required to buy masks and the respondent’s satisfaction with the mask plan. Participants completed the questionnaire between April 24 and April 30, 2020.

## Results

**Figure 1:**
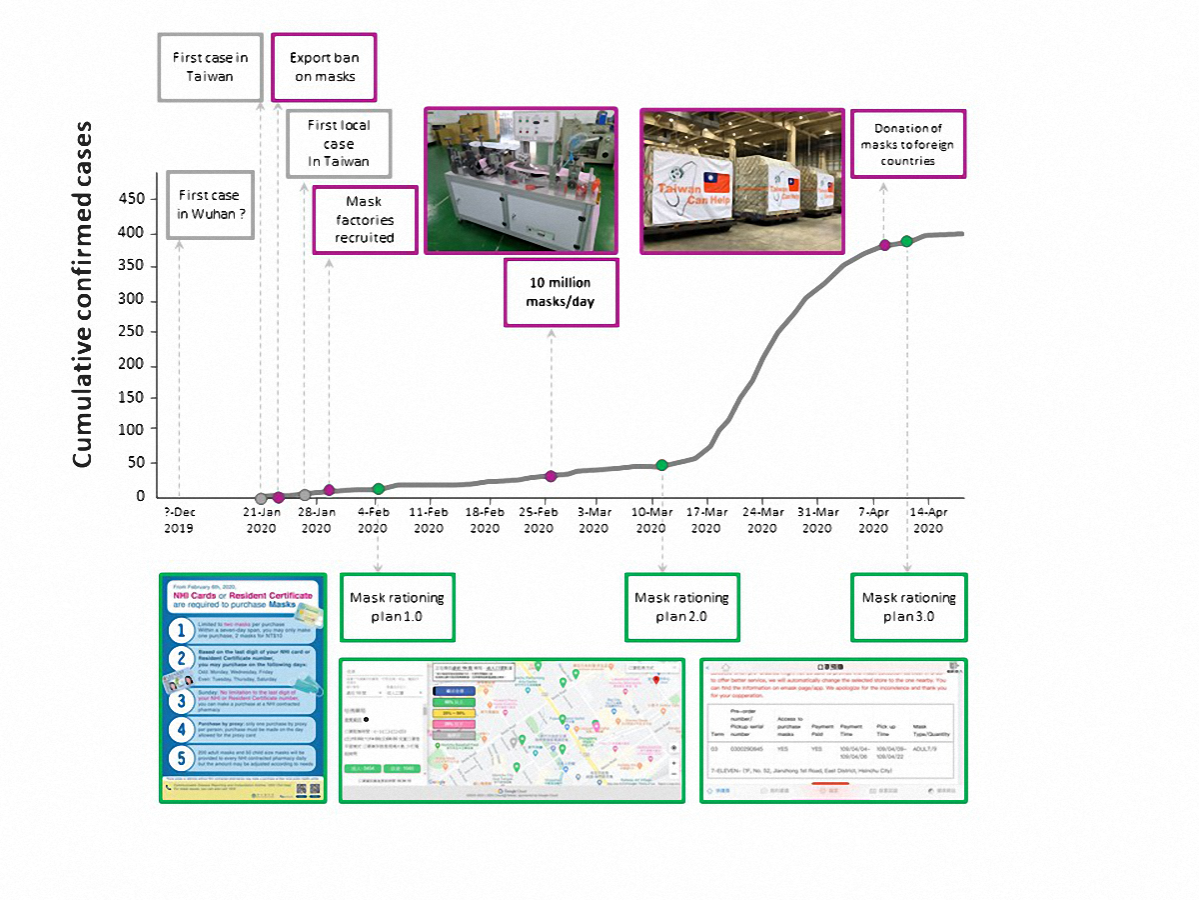
Timeline of confirmed cases in Taiwan and the mask rationing plan. (A) January 21: The first confirmed case in Taiwan. (B) January 24: The first day of the export ban on masks. (C) January 28: The first local case of COVID-19. (D) January 31: Mask factories are identified and provided with mask-making machinery. (E) February 6: Implementation of the mask rationing plan begins. (F) Late February: Mask production was increased from 1 million per day in early February to 10 million per day. (G) March 12: Version 2.0 of the mask rationing plan begins. (H) April 8: Masks are donated to other countries. (I) April 9: Version 3.0 of the mask rationing plan begins.

**Figure 2:**
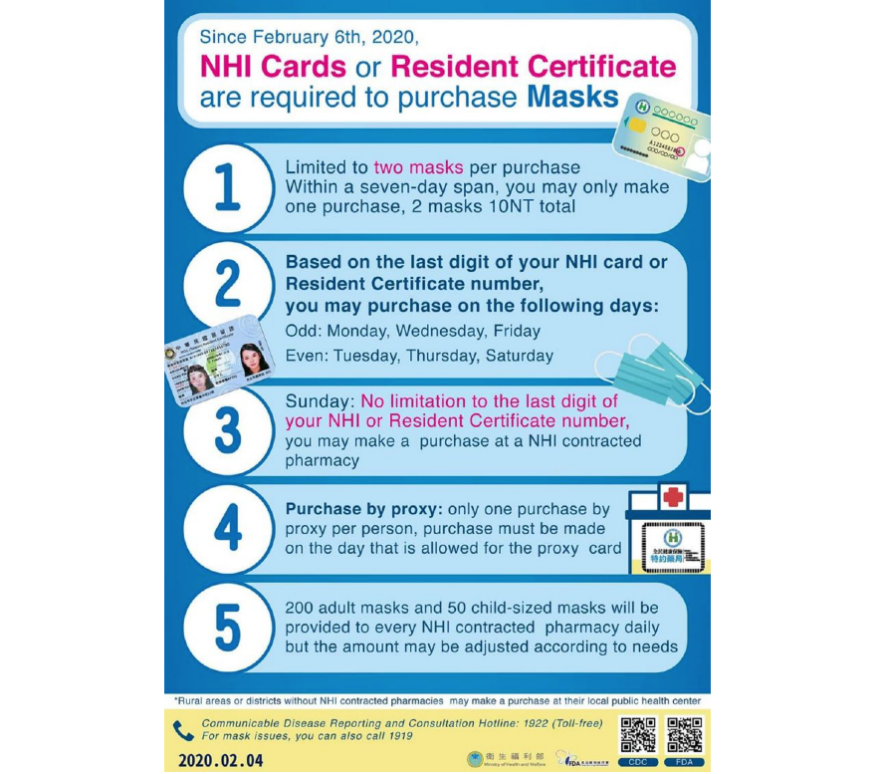
Mask rationing plan 1.0, published on the Ministry of Health and Welfare's Facebook page. Illustration of a new procedure for purchasing medical face masks, which was announced by the Ministry of Health and Welfare of Taiwan on February 6, 2020. Surgical masks can be purchased at local pharmacies upon presentation of a National Health Insurance card. Medical staff are permitted 1-2 masks per day and others can purchase 3 masks per week. A real-time mask map website provided information on mask availability.

**Figure 3:**
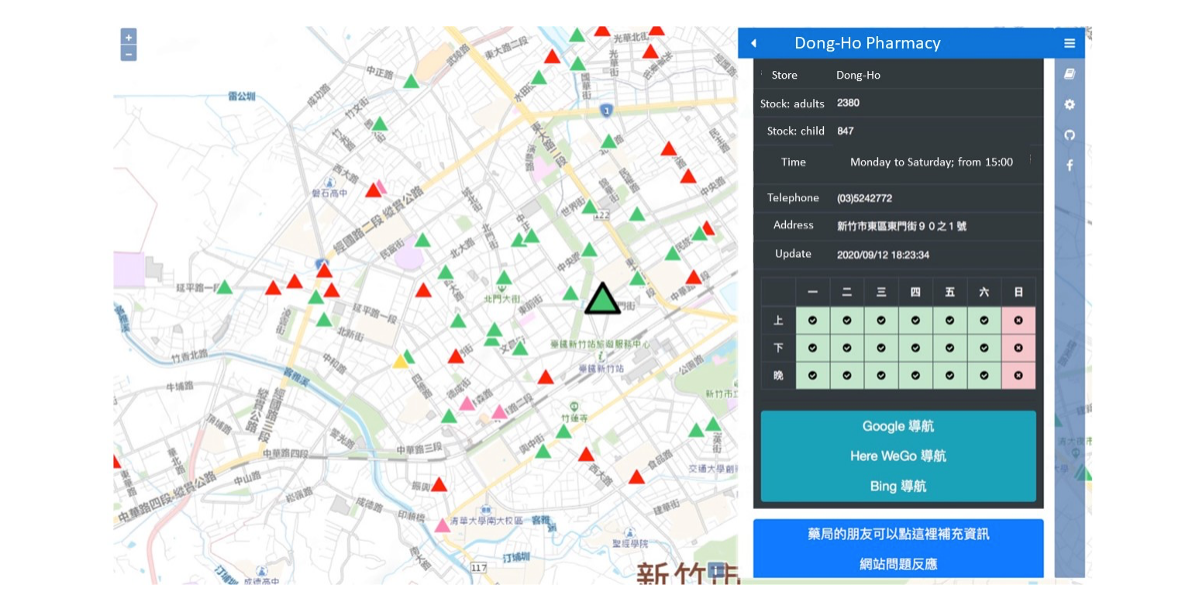
Mask rationing plan 2.0. Screenshot of a map-view software application for face mask availability. Mask purchases can be made by reservation using a mobile app and are available for purchase at convenience stores. Digital mapping software displays information including the pharmacy name, available quantity of adult-sized masks, available quantity of child-sized masks, opening hours, pharmacy phone number, pharmacy address, update time, and opening hours of nearby pharmacies. The triangle colors correspond to the availability of different types of masks or data about masks.

**Figure 4:**
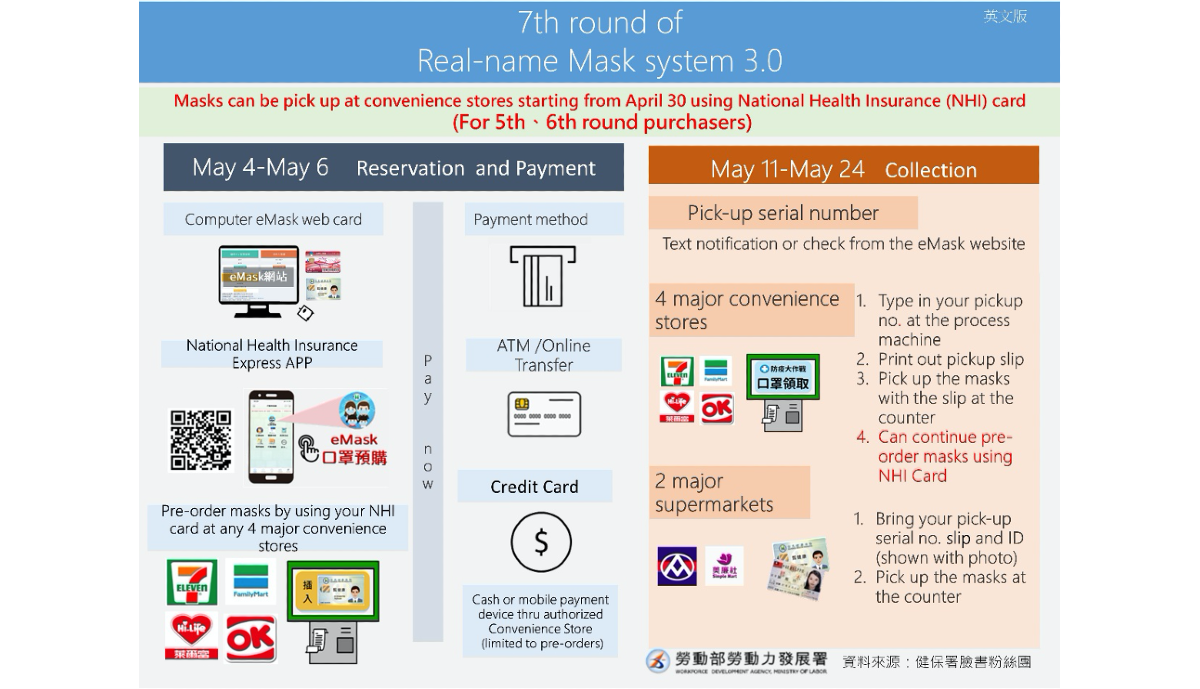
Mask rationing plan 3.0. Adults may purchase 9 masks in a 2-week period, while 10 masks can be purchased for children in a 2-week period. Masks can be purchased via mobile phones or from machines located in convenience stores (the typical transaction time is 1 minute). Masks can be sent abroad.

Figure 1 shows the timeline of Taiwan’s confirmed cases and the evolution of the name-based mask rationing plan. Figures 2-4 show the different versions of the mask rationing plan. The first case of COVID-19 in Taiwan was diagnosed on January 21, 2020, and a “National Mask Team” was formed in late January [5]. All mask factories were recruited and mask machines were provided by the government to ensure Taiwan was able to produce masks quickly. All masks were allocated by the government and people could buy masks at local pharmacies using a unique name-based mask rationing plan. Verification during purchase was required to ensure every resident could buy the masks they needed and to reduce mask stockpiling. Purchases were verified using a national health insurance card and everyone was allowed to buy 2 masks in a 7-day span in early February 2020 (Figure 2). Initially, production capacity was 2 million masks per day in late January 2020. Production capacity increased to 10 million masks per day in late February 2020. The mask plan evolved to 2.0 and real-time mask maps were established to show the availability of masks. Figure 3 shows the user interface of one software application [17] for face mask availability, deployed on websites, social networking sites, and mobile apps in Taiwan [5,18]. As of April 2020, Taiwan had an adequate mask supply and could donate masks to help other countries. The daily production capacity was approximately 16 million masks and people could buy 9 masks during a 14-day period. The mask plan evolved to 3.0 and residents of Taiwan could make online reservations and payments and buy masks at convenience stores (Figure 4) [19].

The questionnaire was administered to 44 adults residing in Taiwan. Table 1 shows the responses of the 44 participants to questionnaires investigating their need for masks and their satisfaction with the mask plan. Most participants agreed that mask wearing is protective (average score of 4.8 [SD 0.47], with 42 participants [95.5%] answering “agree” or “strongly agree”). On average, people needed 1 mask per day (question 8) and the amount allocated per person might be inadequate (average score of 2.07 for question 3 and 2.98 for question 4). Some participants felt anxious if they could not buy an adequate number of masks (score 3.7 [SD 1.22], with 26 participants [59.1%] answering “agree” or “strongly agree”) and the mask plan may have contributed to decreasing this anxiety (score 4.05 [SD 1.15], with 32 participants [72.7%] answering “agree” or “strongly agree”). The average acceptable price was 5.23 NTD (US $0.19) per mask and the price of each mask distributed through the mask plan was 6 NTD. The average amount of time participants were willing to stand in a queue to buy masks was 20.1 (SD 15.2) minutes. In addition, the majority of respondents felt that the mask rationing plan was satisfactory (average score of 4.2 [SD 0.92] points, with 35 participants [79.5%] answering “agree” or “strongly agree”).

**Table 1:** Participant responses to questionnaires investigating the need for masks and their satisfaction with the mask plan (N=44).

Questions	Average response^a^	Participants answering disagree or strongly disagree, n (%)	Participants answering agree or strongly agree, n (%)
1. I believe that wearing a mask may decrease the risk of infection.	4.8 (0.47)	0 (0)	42 (95.5)
2. I feel anxiety if I am unable to buy masks.	3.7 (1.22)	7 (15.9)	26 (59.1)
3. I need only 3 masks for 1 week.	2.07 (1.18)	30 (68.2)	6 (13.6)
4. I need only 9 masks for 2 weeks.	2.98 (1.47)	19 (43.2)	19 (43.2)
5. I feel the price is too high.	2.75 (1.43)	19 (43.2)	11 (25)
6. A mask rationing plan will decrease my anxiety.	4.05 (1.15)	5 (11.4)	32 (72.7)
7. In general, I feel that the mask rationing plan in Taiwan is satisfactory.	4.2 (0.92)	3 (6.8)	35 (79.5)
8. On average, how many masks do you use per day?	1.03 (0.37)	N/A^b^	N/A
9. I think a reasonable price per mask is…	5.23 (2.71) NTD (US $0.19 [$0.1])	N/A	N/A
10. How many minutes would you stand in a queue to buy masks?	20.1 (15.2) minutes	N/A	N/A

^a^Responses to questions 1-7 are presented as mean (SD), where 1=strongly disagree and 5=strongly agree; questions 8-10 were open-ended questions.

^b^N/A: not applicable.

## Discussion

In Taiwan, several strategies were implemented to reduce the spread of COVID-19 and the unique name-based mask rationing plan was believed to play a crucial role in Taiwan’s success in the battle against the virus [5]. This mask plan was executed in January 2020. In this study, we summarized the timeline of the mask plan and introduced some details about clinical practice. Furthermore, we conducted a survey to investigate public responses to the mask plan and whether the mask plan may contribute to decreased anxiety about mask availability.

There have been controversies about the protective effectiveness of masks. In addition, attitudes toward wearing masks have varied across countries. Wearing a face mask is a straightforward and simple measure that the general public can use to prevent the spread of contaminated droplets and limit virus transmission. Specialized medical masks are a critical component of personal protective equipment in clinical settings. However, the effectiveness of mask use among those in the general community remains controversial [7,10,20]. Wearing masks is a common practice in Asian countries, although this is not the case among all cultures [10,21]. Taiwan and some other Asian countries mandated mask wearing in public places, while wearing masks was not mandatory in other countries, such as the United States, Canada, and some European countries. As time went by, mounting evidence demonstrated the benefit of mask wearing for reducing infection spread and it has since become a common recommendation [6]. Additionally, rather than simply wearing a mask due to its potential protective effectiveness, wearing a mask may also be a symbol of safety in some countries [21]. During the COVID-19 pandemic, despite soaring prices, supplies of face masks were rapidly depleted and they quickly became unavailable at stores in the community. Mask stockpiling was not beneficial for infection control and reducing disease spread. In addition, the shortage of personal protective equipment, including masks, among frontline health care personnel also increased the risk of nosocomial infection. To improve the situation, the government of Taiwan implemented a mask rationing plan. The recruitment of all mask factories and an increase in production capacity ensured adequate mask production. The name-based system ensured equitable allocation and people felt less anxious because they could purchase masks. The COVID-19 pandemic is ongoing and this nationwide name-based mask plan may serve as a reference for policy makers worldwide.

The pandemic can cause stress and approximately 1 in 5 COVID-19 survivors experienced mental health problems within 90 days of their COVID-19 diagnosis [22]. The use of masks at the community level may be associated with better mental health [16]. Our questionnaire asked respondents about anxiety related to the face mask shortage at the beginning of the COVID-19 pandemic. People agreed that masks were likely to provide them with effective protection (95.5% [n=42] of participants answered “agree” or “strongly agree”) and more than half of the participants (59.1%, n=26) felt anxious if they were unable to obtain them. However, the required quantities of masks differed among respondents and not everyone was satisfied with the number of masks allocated under the plan (the percentage of participants saying they “disagree” or “strongly disagree” that a given number of masks was adequate was 68.2% [n=30] for question 3 [3 masks in 1 week] and 43.2% [n=19] for question 4 [9 masks in 2 weeks]). The mask rationing plan safeguarded mask purchasing and reduced the public’s perceived anxiety about mask shortages (among 72.7% [n=32] of participants). In a study performed in Poland, Maciaszek et al [23] also indicated an overall decrease in psychopathological symptoms after wearing face coverings in public spaces became obligatory. The mask plan ensured everyone had some masks to wear and this may have had psychological benefits. The implementation of this name-based mask plan and the high uptake of mask wearing may not only have prevented virus transmission, but also decreased anxiety about mask shortages. Although the specific price individuals were willing to pay per mask and acceptable waiting time in queues to purchase masks differed, 4 out of 5 people felt that the mask rationing plan was satisfactory (79.5% [n=35] answered “agree” or “strongly agree”). Further studies are warranted to elucidate the full impact of the mask plan.

While controversy regarding the effectiveness of mask use remains and our study was limited to a small group of responders, the results from this pilot study suggest that the nationwide strategy of mask rationing contributed to the appropriate allocation of masks and a reduction in anxiety about mask shortages. The diagnosis of psychiatric conditions is rigorous and based on the Diagnostic and Statistical Manual of Mental Disorders. We did not aim to confirm a causal relationship between the mask shortage and mental illness; rather, we conducted this preliminary study to indicate whether there is a potential relationship between the mask plan and the public’s perceived anxiety during the pandemic. Further large-scale population-based studies are required to draw stronger conclusions.

In conclusion, the disease burden of the COVID-19 pandemic was still increasing when the name-based mask plan was implemented and the mask plan contributed to the success of Taiwan’s battle against COVID-19. In this study, we highlighted the important timeline dates and components of this mask plan; this may serve as a reference for policy makers. The mask plan safeguarded mask allocation and may also have decreased perceived anxiety during the pandemic. Further studies are required.

## Abbreviations

CDC: Centers for Disease Control, Taiwan
